# Molecular Docking and Anticonvulsant Activity of Newly Synthesized Quinazoline Derivatives

**DOI:** 10.3390/molecules22071094

**Published:** 2017-06-30

**Authors:** Hatem A. Abuelizz, Rabab El Dib, Mohamed Marzouk, El-Hassane Anouar, Yousreya A. Maklad, Hanan N. Attia, Rashad Al-Salahi

**Affiliations:** 1 Department of Pharmaceutical Chemistry, College of Pharmacy, King Saud University, Riyadh 11451, Saudi Arabia; Habuelizz@ksu.edu.sa; 2 Department of Pharmacognosy, College of Pharmacy, King Saud University, Riyadh 11495, Saudi Arabia; reldib@yahoo.com; 3 Department of Pharmacognosy, Faculty of Pharmacy, Helwan University, Cairo 11795, Egypt; 4 Department of Chemistry, College of Science and Humanities, Prince Sattam bin Abdulaziz University, P.O. Box 83, Alkharj 11942, Saudi Arabia; msmarzouk@yahoo.co.uk (M.M.); anouarelhassane@yahoo.fr (E.-H.A.); 5 Chemistry of Natural Products Group, Center of Excellence for Advanced Sciences, National Research Centre, Dokki, Cairo 12622, Egypt; 6 Medicinal and Pharmaceutical Chemistry Department (Pharmacology Group), Pharmaceutical and Drug Industries Research Division, National Research Centre, Dokki, Giza 12622, Egypt; yousreya_maklad@yahoo.com (Y.A.M.); hanan_naeim@yahoo.com (H.N.A.)

**Keywords:** quinazolines, anticonvulsant, phenobarbital, ethosuximide, molecular docking

## Abstract

A new series of quinazoline-4(3*H*)-ones are evaluated for anticonvulsant activity. After intraperitoneal (ip) injection to albino mice at a dose of 100 mg/kg body weight, synthesized quinazolin-4(3*H*)-ones (**1**–**24**) were examined in the maximal electroshock (MES) induced seizures and subcutaneous pentylenetetrazole (scPTZ) induced seizure models in mice. The Rotarod method was applied to determine the neurotoxicity. Most of the compounds displayed anticonvulsant activity in the scPTZ screen at a dose range of 0.204–0.376 mmol/mL. Out of twenty-four, compounds **8**, **13** and **19** proved to be the most active with a remarkable protection (100%) against PTZ induced convulsions and four times more potent activity than ethosuximide. The structure-activity relationship concluded valuable pharmacophoric information, which was confirmed by the molecular docking studies using the target enzyme human carbon anhydrase II (HCA II). The studied quinazoline analogues suggested that the butyl substitution at position 3 has a significant effect on preventing the spread of seizure discharge and on raising the seizure threshold. However, benzyl substitution at position 3 has shown a strong anticonvulsant activity but with less seizure prevention compared to the butyl substitution.

## 1. Introduction

Epilepsy is a ubiquitous disease characterized by chronic and recurrent seizures usually caused by brief and excessive electrical discharges in a group of brain cells [1,2,3]. The World Health Organization (WHO) estimated that almost 80% of the people with epilepsy are living in developing countries and most of them do not receive adequate medication [4]. A wide variety of modalities such as yoga, surgery and drugs have been used for arresting and controlling seizures. Various ketogenic diets have been suggested as useful therapy in pharmacoresistant epileptic patients. This includes classic ketogenic diet, low glycemic index treatment, medium chain triglyceride administration, and a modified Atkins diet [5]. However, drug therapy remains the mainstay in epilepsy treatment. Moreover, remedy is symptomatic in that the available drugs inhibit seizures, but there is no effective prophylaxis or cure is available [6]. A vast number of antiepileptic agents are available to treat various types of seizures aiming to reduce seizure frequency and severity within the scope of a reasonable standard of side effects [7]. For instance, phenytoin, carbamazapine, ethosuccimide and sodium valproate are used to inhibit seizures. However, the ideal anti-seizure drug would suppress all seizures without causing any undesirable effects. Unfortunately, the remedy that is currently used does not only fail to control seizure activity in some patients, but it frequently remains inadequate and the patients suffer from a lot of specific problems like depression, neurotoxicity, sedation, ataxia, and hypnosis [7]. The currently used antiepileptic drugs abolish only the ictal but not the interictal epileptiform discharges. However, clinical evidence indicates that antiepileptic drugs are not affecting the interictal activity which is the activity that effectively stop seizures [8]. The undesirable side effects of the clinically used drugs often render therapy difficult; so there is high demand for new anticonvulsants [7].

The carbonic anhydrase inhibition (CA; EC 4.2.1.1) has demonstrated interesting pharmacologic applications such as anti-convulsant, anti-glaucoma and anti-cancer agents. These enzymes have shown a potential target for designing anti-convulsant drugs with a novel mechanism of action. The enzymes play an important role in the anion exchange processes [9]. 16 CA isozymes have been described to date in mammals and among them as catalysts for carbon dioxide hydration is CA II. This will allow the maintenance of pH homeostasis in the human body. It was reported that a series of quinazoline derivatives were designed and synthesized as potential CA inhibitors [10]. Moreover, a literature survey reveals that the chemistry of quinazolines and their derivatives has received considerable attention owing to their synthetic and effective anticonvulsant importance [11,12,13,14,15,16,17,18,19,20,21,22]. Methaqualone and mecloqualone (**I**, **II**), as quinazoline analogues, approved as anticonvulsant drugs [1] and the 6-chloro derivative of (**I**) was found to possess remarkable anticonvulsant potency against electroshock-induced convulsions (1.5 times than phenytoin sodium) and against pentylenetetrazol (PTZ)-induced seizures (10 times than troxidone) [17]. Over the years, medicinal chemists have modified and substituted different chemical moieties at position 3 of 4(3*H*)-quinazoline to yield structural analogues with potent anticonvulsant activity. The recent reported quinazoline analogues (**III**, **IV**) possess significant anticonvulsant activities (ED_50_ = 73.1 and 11.79 mg/kg) and showed a 100% protection against PTZ induced clonic convulsion [16,17].

**Figure 1:**
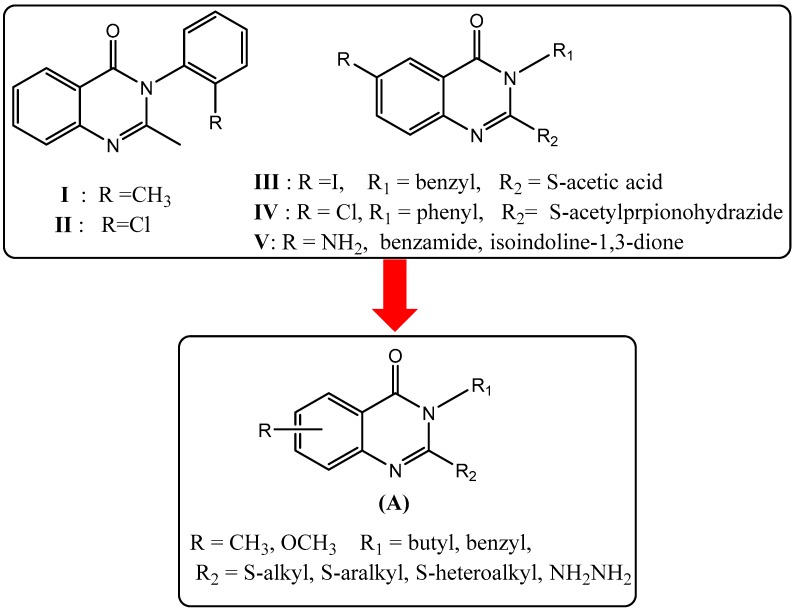
The structures of anticonvulsant and designed quinazolines.

Based on the aforementioned considerations, bearing in mind the inherited anticonvulsant potency of quinazoline nuclei together with the similarities between methaqualone, mecloqualone, the reported anticonvulsants and our synthesized derivatives (**A**) (Figure 1), we report the evaluation of a new synthesized quinazolines series (**1**–**24**) [23,24,25] for anticonvulsant effects to explore the influence of butyl and benzyl groups at position 3 of the quinazolines (**4**–**16,22,24**) and (**16**–**21**,**23**), respectively. In addition, this study aims to investigate the electronic effect of the electron donating- and withdrawing groups at position 2- on the anticonvulsant activity and hence deduce the structure activity relationship (SAR).

## 2. Results and Discussion

### 2.1. Anticonvulsant Activity

**Scheme 1:**
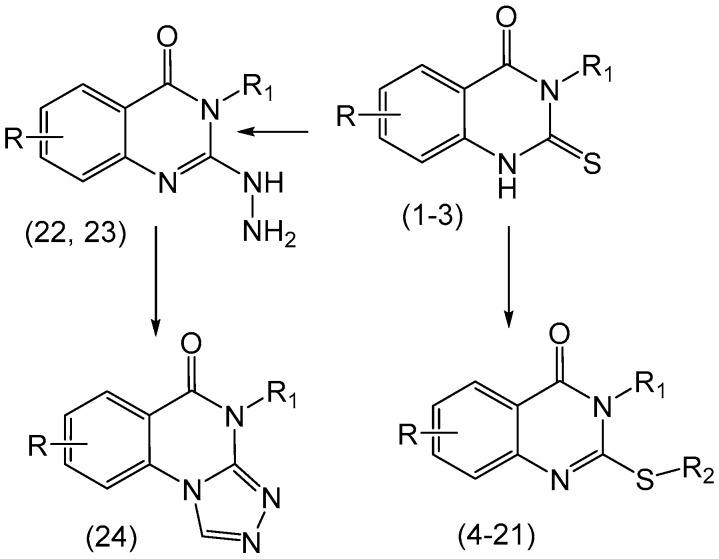
Synthetic pathways for the quinazolines **1**–**24**.

As shown in Scheme 1 and Table 1, the synthetic pathways and full physicochemical characterization of compounds **1**–**24** have been described [23,24,25].

**Figure 2:**
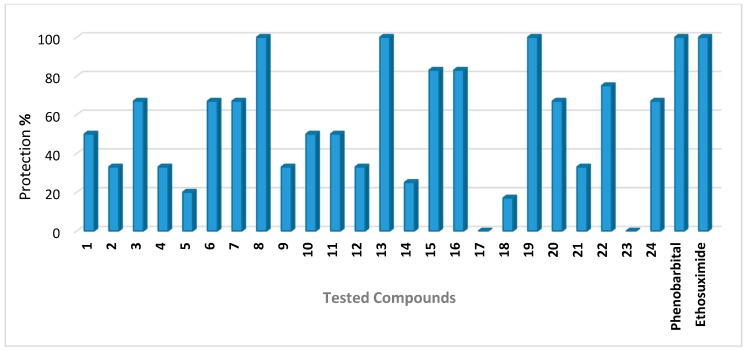
The scPTZ protection activity of the synthesized compounds **1**–**24** compared with phenobarbital and ethosuximide as reference drugs.

Animal seizure models are well known predictable techniques for the discovery of novel bioactive chemical agents for the preclinical treatment of epilepsy. These models cover diverse classes of epilepsy with different categories [26,27]. Early stages of anticonvulsant testing are feasible via the acute seizure model, induced by either electric (MES) or chemical (scPTZ) stimulus in normal animals, which are also labeled as the “gold standard screen tests” [28]. The anticonvulsant drug development (ADD) program protocol of National Institute of Health, provided by the epilepsy section of National Institute of Neurological Disorders and Stroke (NINIDS), has been adopted in the current investigation. The scPTZ screening test utilizes chemical stimuli to induce myoclonic seizures, simulating human generalized absence seizures (petit mal epilepsy) [28,29,30]. It is worth noting that this model simulates human tonic-clonic epilepsy and partial convulsions, with or without secondary generalization. Meanwhile, a MES test is conducted using electrical stimulus to induce generalized tonic-clonic seizures (grand mal epilepsy) and screen compounds that prevent seizure spreading. In the present investigation, test compounds **1**–**24** were injected at doses of 100 mg/kg (ip) and PTZ was used at a dose level of 85 mg/kg (sc), 45 min after the test compounds. This dose was found to be the minimum dose that induced 100% clonic convulsion. Any of the tested compounds that were able to prevent such episodes or that delayed this time pattern in varying magnitude will be considered to possess an anticonvulsant activity. Target compounds **1**–**24** have been initially evaluated by the scPTZ test, and candidates that raised seizure threshold and provided 100% protection were further tested by the MES screen. Additionally, all candidate compounds underwent an acute neurological toxicity test using the rotarod apparatus to identify minimal motor impairment. Anticonvulsant activity of the new candidates **1**–**24** were evaluated 45 min following compound administration. Results of the primary (phase-I) screening expressed as % protection of the tested products **1**–**24**, as well as neurotoxicity are shown in Table 2. The obtained findings revealed that the initial evaluation of most candidates showed variable anticonvulsant activity ranging from 17–100% protection against scPTZ-induced seizures at the tested dose level equivalent to 100 mg/kg. Data obtained indicated that compounds **8** (0.248 mmol/kg), **13** (0.239 mmol/kg) and **19** (0.338 mmol/kg) were the most potent congeners exhibiting 100% protection against myoclonic seizures without neurotoxicity. In addition, compounds **8**, **13** and **19** were more potent than ethosuximide by 4.3, 4.4, 3.13 folds, respectively, (3–4 folds). Meanwhile, the reference drugs phenobarbital and ethosuximide exhibited 100% protection at dose levels 0.13 and 1.06 mmol/kg, respectively. Moreover, compounds **16** (0.252 mmol/kg) and **15** (0.245 mmol/kg) exhibited an equipotent effect (83% protection) at the tested dose levels, which was almost four folds more potent than ethosuximide. On the other hand, compounds **17** and **23** lacked anti-seizure property at dose levels 0.329 and 0.291 mmol/kg, respectively. The remaining compounds exerted variable anticonvulsant potential ranging from 20% to 75% protection. Candidate compounds in this series are arranged according to the potency of raising seizure threshold in the following descending order: **8** = **13** = **19** > **15** = **16** > **22** > **3** = **6** = **7** = **20** = **24** > **1** = **10** = **11** > **2** = **4** = **9** = **12** = **21** > **14** > **5** > **18** (Figure 2).

**Figure 3:**
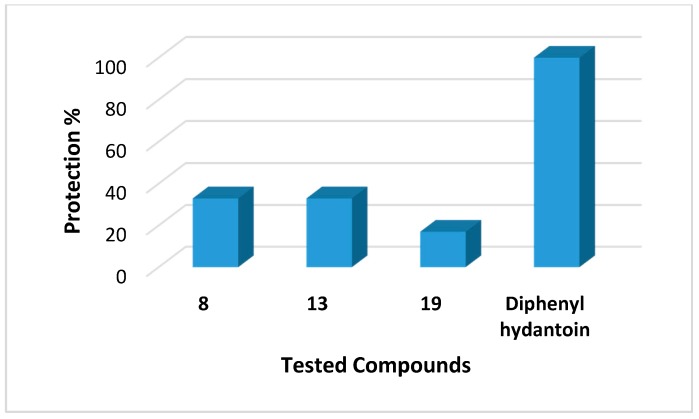
MES-induced seizure protection activity of the synthesized compounds **8**, **13** and **19** compared with diphenylhydantoin as a reference drug.

Furthermore, compounds **8**, **13** and **19** subjected to MES-induced seizure and produced 17, 33 and 33% protection against MES-induced seizure (Figure 3), respectively. These findings suggest that **8**, **13** and **19** possess the ability to prevent the spread of seizure discharge and to raise seizure threshold. In the current investigation, all candidates **1**–**24** were devoid of neurotoxicologically effects (motor dysfunction), which has detected by using the rotarod screen [31].

Based on the results in Table 2, structure activity correlation of the compounds **1**–**24** netted some valuable pharmacophoric information about the anticonvulsant activity. The parent compounds **1**, **2** and **3** exhibited anticonvulsant activity (50%, 33%, 67%, respectively), however, compound **3** appeared stronger than **1** and **2**. The increase in activity may be due to the fact that compound **3** has a highly lipophilic group (butyl) that enhanced such activity. A variation in the anticonvulsant activity was noticed with chemical transformation of **3** into **4**–**15**, **22** and **24**. Compounds **4**, **5**, **9**, **10**–**12** and **14** showed low activity; **8**, **13** and **15** exhibited the highest activity in relation to the parent **3**.

In regards to the benzyl substitution at position 3, compound **21** has shown the same activity as its parent **2** (33%), however, compound **20** demonstrated potent activity (67%) and compounds **16** and **19** showed the highest activity in regards to **1**.

It can be concluded that variation and configuration of substituents on S-benzyl ring affected the anticonvulsant activity. This is seen in the increment of anticonvulsant activity in compounds **8** and **13** containing electron withdrawing (Cl) and electron donating (OCH_3_) groups, in which both compounds showed the 100% protection against scPTZ-induced seizures. This indicates that the presence of Cl group in **8** enhanced the lipophilicity, which positively affected the anticonvulsant activity. Whereas, the presence of an electron donating group was beneficial for the efficiency observed in compound **13**.

Incorporation of heteroalkyl groups into **1** and **3** afforded **19** and **15** with highly increase in the protection against scPTZ-induced seizures. This suggests that the presence of benzodiazoxazole and morphilinoethyl moieties might play a substantial role in the anticonvulsant activity. Moreover, compound **19** showed a high protection activity against scPTZ-and MES-induced seizures protection activity than **15** (Figure 3). Hydrazinolysis of **1** and **3** produced the anticonvulsant active **22** (75%) and inactive **23** (0%) products.

Furthermore, it was found that cyclization of **22** into corresponding triazole **24** was not found to be fruitful as the presence of this five membered ring and showed a slight decrease in the percentage of protection against seizure (67%).

### 2.2. Molecular Docking

A series of aromatic/heterocyclic compounds were prepared to design carbonic anhydrase inhibitors. In 2009, a group of isoquinoline derivatives were prepared by Rosaria Gitto and evaluated against several mice CA isoforms. Some of them have shown weak anticonvulsant activity with low CA selectivity [32]. However, in 2009, De Simone examined these derivatives against all mammalian carbonic anhydrases. Strong inhibition of CA II, VII, IX, and XII were correlated with sufficiently high liposolubility [33]. CA II controls the bicarbonate-mediated virulence induction and the conversion of CO_2_ into bicarbonate, which plays an important role in virulence induction. hCA II x-ray crystal structure in complex with dithiocarbamate was reported. The amino acid residues involved in the binding were Asn67, Gln92, Phe131, Leu198 and Pro202. This finding provided a deep understanding at the atomic level of the interactions between the enzyme and the inhibitor [34].

**Figure 4:**
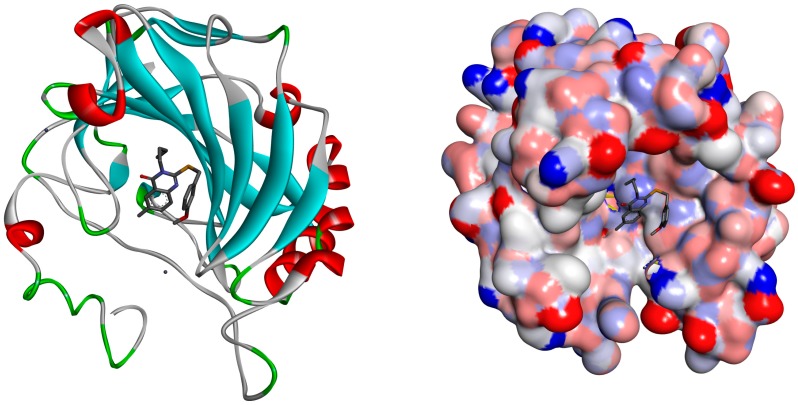
Docking of quinazoline **13** in the active site of hCA II (**left**), and the electrostatic surface potential of hCA II in complex with **13** (**right**).

**Figure 5:**
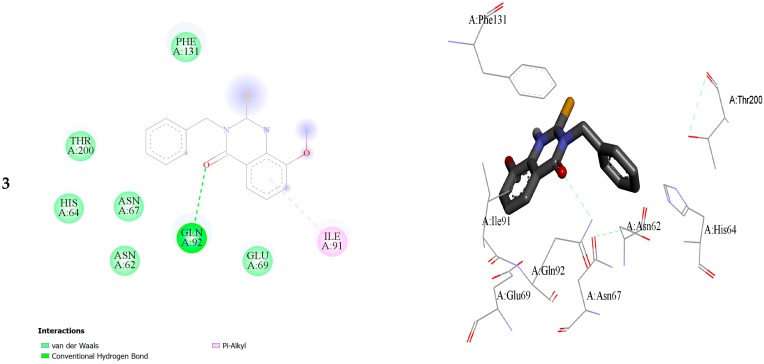

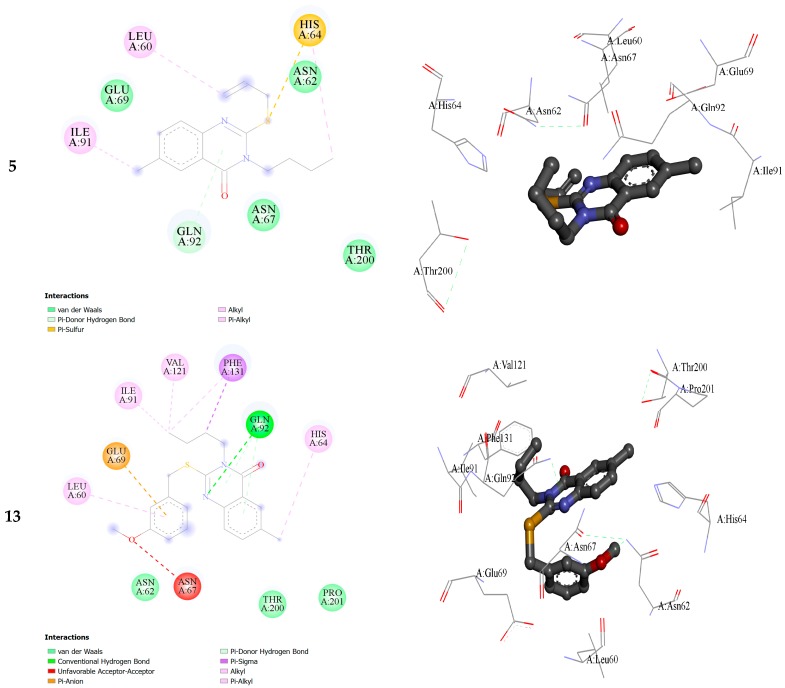
2D and 3D closest interactions between hCA II and quinazolines **3**, **5** and **13** when bound to the enzyme.

Docking analyses were performed to obtain more insights into the binding mode of docked ligands and the active site of hCA II isomer, i.e., the investigation of interaction of the high (**13**), moderate (**2**) and low anticonvulsant (**5**) synthesized quinazoline derivatives to the active site hCAII. The scores of docking results are based on the free binding energies and the hydrogen bond interactions involved in the binding mode. The obtained docking scores of results of low (**5**), moderate (**3**) and high (**13**) synthesized derivatives are presented in Table 3. The docked quinazoline **13** in the active binding site is shown in Figure 4. The closest interactions between quinazolines **3**, **5** and **13** with the residue of hCA II are shown in Figure 5.

The variation of binding energy of the stable enzyme-ligand complexes is in good agreement with the observed anticonvulsant activities of quinazoline derivatives. Indeed, quinazoline **13** exhibited the highest activity with the lowest energy of −4.01 kcal/mol among the three docked quinazolines. However, the less active one, **5**, possesses the highest energy (−3.19 kcal/mol). In addition, the higher anticonvulsant activity of quinazoline **13** compared to quinazolines **3** and **5** may also refer to the number of established hydrogen bonding in enzyme-ligand complexes. On one hand, two hydrogen bonds are formed between **13** and amino acid residues of hCA II active site Asn62 and Gln92 (see Table 3 and Figure 5). On the other hand, only one hydrogen is formed in case of **3** in the active site with the amino acid residue Gln92. However, no hydrogen bonds are formed between quinazoline **5** and the closest amino acid in the active site of hCA II. Moreover, compound **13** binds to 10 amino acid residues at the active site compared to compound **5**, which exhibited only 5 binding amino acid residues and compound **3** with only 4 amino acids binding. This may support the notion that the high activity presented by compound **13** is because of the higher number of hydrogen bonds and binding amino acid residues at the hCA II active site. The docking of the selected quinazoline derivatives reveal that they bind to the active site of hCA II in a similar manner to the previous reported docking studies [35,36].

## 3. Materials and Methods

### 3.1. Animals

Adult male Swiss albino mice weighing 18–25 g were used in the current study. They were obtained from Animals House Colony of the National Research Centre, Cairo, Egypt. The animals were housed under standardized conditions (room temperature 23 + 2 °C; relative humidity 55 + 5%; 12 h-light/dark cycle) and had free access to tap water and standard rat chow throughout the entire experimental period. Procedures involving animals and their care were performed after the ethics committee of the National Research Center (16090) and in accordance with the recommendations for the proper care and use of laboratory animals “Canadian Council on Animal Care Guidelines, 1984”. Extensive efforts were made to minimize animal suffering and the proper number of animals were used to obtain reliable data.

### 3.2. Drugs and Chemicals

Diphenylhydantoin (Nasr Co., Giza, Egypt), Ethosuximide (Pfizer Co., Giza, Egypt), Phenobarbital (Memphis Co. for Pharmaceutical and Chemical Industries, Cairo, Egypt). Tween-80 and Pentylenetetrazol were purchased from Sigma (St. Louis, MO, USA). The reference drugs and tested compounds were suspended in 7% tween-80 and administered intraperitoneal at a volume of 0.1 mL/10 g body weight.

### 3.3. Methods

One week following adaptation to laboratory conditions, the animals were randomly assigned to control and experimental groups consisting of six mice each.

#### 3.3.1. Subcutaneous Pentylenetetrazol (scPTZ) Screen

This test produces threshold or minimal (clonic) seizures. Subcutaneous (sc) injection of aqueous solution of PTZ at a dose of 85 mg/kg was administered in the loose fold of skin at the back of the mouse neck. This dose (called convulsive dose 97 (CD97)), causes seizures in more than 97% of animals [37]. The test is carried out by giving scPTZ, 45 min after intraperitoneal injection (i.p.) of the test compounds at a dose level equivalent to 100 mg/kg. The tested animals were closely monitored for the occurrence of seizures for 30 min. A threshold convulsion was defined as one episode of clonic convulsions, which persisted for at least 5 s. Absence of a single 5 s episode of clonic spasms during the 30 min period of observation is taken as the end point in this test [28,38].

#### 3.3.2. Maximal Electroshock Seizure (MES) Screen

Electroconvulsions were induced by a fixed current intensity of 25 mA and 0.2 s stimulus duration, which was delivered via ear clip electrodes using a Rodent Shocker generator (constant-current stimulator Type 221, Hugo Sachs Elektronik, Freiburg, Germany), forty-five min following i.p. injection of the test compound. The criterion for the occurrence of seizure activity was the tonic hind limb extension (i.e., the hind limbs of animals outstretched 180° to the plane of the body axis) [39].

#### 3.3.3. Neurotoxicity

The neurotoxicity of the animals was evaluated by adopting the rotarod test [31]. In this test, the animals were pre-trained to maintain equilibrium on a rotating 1 inch diameter knurled plastic rod (rotarod, UGO Basile, 47600, Varese, Italy) at a fixed speed of 10 rpm for 60 s in each of the three trials. Only animals that fulfill this criterion were included in the experiment. The animals in the experimental groups (*n* = 6) were given an ip injection of one of the test compounds. Forty-five min later, the mice were placed again on the rotating rod and the motor performance time was monitored for up to 60 s. Neurotoxicity was indicated by the inability of the animal to maintain equilibrium on the rod for at least 60 s.

### 3.4. Molecular Docking

It is reported that the anticonvulsant, antepileptic and antiseizure drugs may account for hCA inhibition [35,36]. The choice of consistent hCA enzyme was based on reported litterature [40]. Hence, X-ray coordinates of the hCA II isomer was taken from the RCSB data bank (PDB file with code 3F8E), in which, *cis*-2-hydroxy-4-(1*S*-3–methylbutyl)-3-methoxy-cinamic acid perfectly fits with the active site of [41]. Water molecules were removed, polar hydrogen atoms and Kollman charge were added to the enzyme structure using the automated tool in AutoDoc 4.2.6 (The Scripps Research Institute (TSRI), La Jolla, CA, USA) [42]. The active site was identified based on co-crystallized ligand structure in the addcuct structure (i.e., enzyme-ligand complex) [41]. The docking study was validated by re-docking of the original ligand into the enzyme active sites, that lead to an RMSD value less than 2 Å. The optmization of quinazoline derivatives geometries (**1**–**24**) was carried out by using molecular mechanics force field MM^+^ as implemented in HyperChem^TM^ program Release 7.52 for windows (Hypercube, Inc., Gainesville, FL, USA). The optimized structures were saved as pdb files. Nonpolar hydrogens were merged and rotatable bonds were defined for each ligand. Docking studies were performed by Lamarckian genetic algorithm, with 500 as the total number of run for each binding site. In each respective run, a population of 150 individuals with 27,000 generations and 250,000 energy evaluations were employed. Operator weights for crossover, mutation, and elitism were set to 0.8, 0.02, and 1, respectively. The binding site was defined using a grid of 20 × 20 × 20 points each with a grid space of 0.375 Å centered at coordinates *x* = −5.095, *y* = 6.414, and *z* = 9.427. The calculation have been carried out using an Intel (R) Core (TM) i5-3770 CPU @ 3.40 GHz workstation (Windows version, Dell Inc., Round Rock, TX, USA). Cluster analyses were performed on docked results using a root mean-square deviation (RMSD) tolerance of 2.0 Å. The protein-ligand complexes were visualized and analyzed using AutoDockTools and Discovery Studio version 4.0 (Accelrys Software Inc., San Diego, CA, USA).

## 4. Conclusions

The results revealed that most of the compounds displayed 17–100% anticonvulsant activity in the scPTZ screen at a dose range of 0.204–0.376 mmol/kg. Compounds **8**, **13** and **19** appeared the most potent in this series at doses of 0.248, 0.239 and 0.338 mmol/kg, respectively. They were 3–4 fold more potent than ethosuxmide (1.06 mmol/kg) and less than phenobarbital (0.13 mmol/kg). The butyl substitution at position 3 plays a significant role in the anticonvulsant activity and the seizure spreading, which presented in compounds **8** and **13**. The benzodiazoxazole moiety at position 2 enhances the anticonvulsant activity of the benzyl substituted compounds at position 3 with less seizure prevention as in the case of compound **19**. Moreover, the methyl substituted quinazoline ring displayed a stronger anticonvulsant activity than the methoxy substitution. Molecular docking results showed that the high anticonvulsant potency of the synthesized quinazolines is mainly due to the lowest binding energy of the stable complex ligand-enzyme, as well as the number of hydrogen bonds and intermolecular amino acids binding at the active site of hCA II.

## Figures and Tables

**Table 1:** Synthesized quinazoline derivatives **1**–**24**.

Compounds	R	R_1_	R_2_
**1**	methyl	benzyl	-
**2**	methoxy	benzyl	-
**3**	methyl	butyl	-
**4**	methyl	butyl	ethyl
**5**	methyl	butyl	allyl
**6**	methyl	butyl	2-Me-benzyl
**7**	methyl	butyl	3-Me-benzyl
**8**	methyl	butyl	4-Cl-benzyl
**9**	methyl	butyl	4-NO_2_-benzyl
**10**	methyl	butyl	2-CN-benzyl
**11**	methyl	butyl	3-CN-benzyl
**12**	methyl	butyl	4-CN-benzyl
**13**	methyl	butyl	3-methoxy-benzyl
**14**	methyl	butyl	(1*H*-Benzoimidazol-2-yl)methyl
**15**	methyl	butyl	morphilinoethyl
**16**	methyl	benzyl	4-CN-benzyl
**17**	methyl	benzyl	2-methyl-benzyl
**18**	methyl	benzyl	4-NO_2_-benzyl
**19**	methyl	benzyl	7-NO_2_-benzoxadiazole
**20**	methoxy	benzyl	3-(Phthalimido-2-yl)propyl
**21**	methoxy	benzyl	morphilinoethyl
**22**	methyl	butyl	hydrazine
**23**	methyl	benzyl	hydrazine
**24**	methyl	butyl	-

**Table 2:** Anticonvulsant activity and neurotoxicity of compounds **1**–**24** relative to the reference standards against both pentylenetetrazol and electro-induced seizures in adult male albino mice.

Compounds	Dose ^a^		% Protection		Neurotoxicity ^b^
	(mg/kg)	(mmol/kg)	scPTZ	MES	
**1**	100	0.301	50	Nd	0/6
**2**	100	0.376	33	Nd	0/6
**3**	100	0.234	67	Nd	0/6
**4**	100	0.239	33	Nd	0/6
**5**	100	0.253	20	Nd	0/6
**6**	100	0.267	67	Nd	0/6
**7**	100	0.212	67	Nd	0/6
**8**	100	0.248	100	33	0/6
**9**	100	0.241	33	Nd	0/6
**10**	100	0.204	50	Nd	0/6
**11**	100	0.255	50	Nd	0/6
**12**	100	0.355	33	Nd	0/6
**13**	100	0.239	100	33	0/6
**14**	100	0.373	25	Nd	0/6
**15**	100	0.245	83	Nd	0/6
**16**	100	0.252	83	Nd	0/6
**17**	100	0.329	0	Nd	0/6
**18**	100	0.309	17	Nd	0/6
**19**	100	0.338	100	17	0/6
**20**	100	0.331	67	Nd	0/6
**21**	100	0.251	33	Nd	0/6
**22**	100	0.352	75	Nd	0/6
**23**	100	0.291	0	Nd	0/6
**24**	100	0.325	67	Nd	0/6
Phenobarbital	30	0.130	100	Nd	Nd
Ethosuximide	150	1.060	100	Nd	Nd
Diphenyl Hydantoin	45	0.160	-	100	Nd

**^a^** The minimal dose that exerted the maximal anticonvulsant potential; **^b^** Rotarod test: number of animals with neurotoxicity/total number of tested animals; Nd: not determined, - indicates the absence of anticonvulsant activity at the tested dose level.

**Table 3:** Docking binding energies and binding interactions of quinazoline derivatives **3**, **5** and **13**.

Within the Active Binding Site of hCA II Isomer. Quinazoline Derivatives	Free Binding Energy (kcal/mol)	H-Bonds (HBs)	Closest Interaction of Quinazoline and Active Site Residues
**3**	−3.55	One HB: (GLN92-Q3)	ASN67, HIS64, GLN92, ILE 91
**5**	−3.19	One HB: (GLN92-Q5)	HIS64, ASN62, ASN67, GLU69, GLN92
**13**	−4.01	Two HBs ASN62-Q13 GLN92-Q13	ILE91, PHE131, GLU69, ASN62, LEU60, ASN67, GLN92, THR200, HIS64, PRO201
